# Stress in hospital nurses: association between coping strategies and sociodemographic and occupational variables

**DOI:** 10.1590/0034-7167-2025-0258

**Published:** 2026-07-06

**Authors:** Manuela Ribeiro dos Santos, Daiani Modernel Xavier

**Affiliations:** I Universidade Federal do Rio Grande. Rio Grande, Rio Grande do Sul, Brazil

**Keywords:** Stress, Psychological, Adaptation, Psychological, Interpersonal Relations, Occupational Stress, Nursing., Estrés Psicológico, Adaptación Psicológica, Relaciones Interpersonales, Salud Laboral, Enfermería.

## Abstract

**Objectives:**

to evaluate the associations between nurses’ stress levels, coping strategies, and sociodemographic and occupational variables among hospital nurses.

**Methods:**

this descriptive, cross-sectional, quantitative study included 124 nurses from a university hospital in southern Brazil. Data were collected using self-administered instruments: a sociodemographic questionnaire, the Brazilian versions of Work Stress Scale, and the Work Coping Response Inventory. Data analysis was conducted using descriptive and inferential statistics.

**Results:**

stress levels ranged from low to moderate and high. Nurses with higher stress levels more frequently used escape-avoidance coping strategies, whereas those with lower stress levels reported better self-perceived mental health.

**Conclusions:**

the findings support the development of institutional programs and the strengthening of comprehensive occupational health care guidelines within the Unified Health System.

## RESUMEN

**Objetivos:**

evaluar la asociación entre el nivel de estrés de enfermeros hospitalarios, las estrategias de *coping* utilizadas y las variables sociodemográficas y ocupacionales.

**Métodos:**

estudio descriptivo, transversal, de abordaje cuantitativo, realizado con 124 enfermeros de un hospital universitario en el sur de Brasil. Se utilizaron instrumentos autoaplicables: cuestionario sociodemográfico, Escala de Estrés en Trabajo e Inventario de Estrategias de *Coping*. El análisis de los datos fue realizado por medio de estadística descriptiva e inferencial.

**Resultados:**

se verificaron niveles de estrés, en nivel bajo de estrés con proximidad a los niveles medio y alto. Enfermeros con estrés elevado utilizaron la estrategia de *coping* del tipo Fuga y Esquivar, en cuanto aquellos con bajo estrés relataron mejor autopercepción de la salud mental.

**Conclusiones:**

los hallazgos contribuyen para auxiliar programas institucionales y reforzar directrices de atención integral a la salud del trabajador en el Sistema Único de Salud.

## INTRODUCTION

The work of nurses in hospital settings is characterized by high complexity, involving multiple technical, emotional, and organizational demands that require professionals to constantly adapt and respond to high-pressure situations^(1,2)^. Such a context makes the profession particularly susceptible to occupational stress, which results from the interaction between workplace demands and the perception of individual resources available to cope with them^(3-5)^. In this context, coping strategies-understood as cognitive and behavioral efforts to adapt to adversity-play a central role, as they can either mitigate or intensify the negative effects of stress depending on their adaptive or dysfunctional nature^(6,7)^.

The relational and care-based nature of the profession, combined with frequent exposure to human suffering, death, and institutional constraints, contributes to the development of occupational stress^(3)^. When not properly managed, this phenomenon compromises not only the physical and mental health of professionals but also the quality of care delivered to the population.

Occupational stress is a multifactorial phenomenon that results from the interaction between workplace demands and professionals’ perception of the individual resources available to cope with them^(4,5)^. In nursing, coping strategies emerge as key mechanisms for managing stress. Adaptive strategies, such as problem solving, emotional self-regulation, and seeking social support, are effective in mitigating the negative effects of stress and strengthening resilience. Conversely, the recurrent use of dysfunctional strategies, including escape, avoidance, and emotional withdrawal, tends to exacerbate psychological distress and increase vulnerability to illness^(6,7)^.

In their daily efforts to manage the pressures and demands of work, nurses may exhibit behaviors such as social withdrawal, irritability, lack of motivation, procrastination, cynicism, and interpersonal conflict. These manifestations reflect ineffective coping strategies for occupational stress and contribute to its perpetuation. When not properly addressed, such behaviors compromise the organizational climate, weaken team cohesion, and negatively affect patient safety, representing a significant public health concern in hospital settings^(8)^.

In healthcare settings, nurses often face work environments characterized by hostility, excessive workload, and high levels of stress. This context is associated with an increased incidence of burnout, depressive symptoms, absenteeism, and intention to leave the profession^(9)^. In light of this scenario, it is essential for healthcare institutions to implement measures that promote healthy work environments, foster positive coping strategies, and enable the early identification of signs of psychological distress as an integral component of employee care policies^(10)^.

Furthermore, organizational interventions that combine emotional support strategies, training in adaptive coping strategies, and the strengthening of social support networks show promising results in reducing stress and promoting mental health among professionals. Structured emotional support programs in the workplace contribute to lowering stress prevalence and enhancing psychological well-being^(11,12)^. These interventions directly impact the quality of care delivered and the sustainability of healthcare services.

It is crucial to recognize that nurses have different ways of coping with occupational stress, as their responses reflect a complex interaction between individual and contextual factors. Sociodemographic aspects-such as age, gender, marital status, and education-and occupational variables, including weekly working hours, professional experience, work sector, and type of employment relationship, directly influence the perception of stress and the selection of coping strategies^(13-15)^. These factors function as social determinants of worker health and must be considered critically, as they contribute to the development of different psychosocial vulnerability profiles in hospitals^(16)^. Therefore, recognizing these variables is essential for developing equitable and effective mental health interventions and promoting healthy work environments.

Given the complexity and magnitude of this topic, it is crucial to deepen the understanding of the interactions between occupational stress, coping strategies, and perceptions of mental health in nursing. Recognizing these relationships is essential to guiding management practices, institutional policies, and actions aimed at preventing mental illness. This study is particularly relevant as it highlights coping patterns and their effects on professionals’ well-being, contributing to the advancement of interventions in occupational health.

In this sense, this study aims to answer the following guiding question: How is the level of stress among hospital nurses associated with coping strategies and sociodemographic and occupational variables?

## OBJECTIVES

To evaluate the association between hospital nurses’ stress levels, coping strategies, and sociodemographic and occupational variables.

## METHODS

### Ethical Aspects

The study was approved by the Institutional Review Board, in accordance with Resolution 466/2012 of the National Health Council^(17)^. Data collection began after receiving ethical approval and institutional authorization. In compliance with the principle of autonomy, all participants were informed about the study procedures and signed two copies of the Free and Informed Consent Form, one of which was provided to each participant.

### Study design and sampling

This was a descriptive-exploratory, cross-sectional study with a quantitative approach. This design aimed to describe and deepen understanding of underexplored phenomena by observing a population at a single point in time and applying statistical analyses to identify patterns and relationships^(18)^. The study was conducted at a public university hospital, affiliated with the Unified Health System (SUS), located in the far south of Brazil. The institution serves as a regional reference center for mediumand high-complexity care and has a comprehensive care structure. Nurses working in the internal medicine, surgical unit, traumatology, maternity, pediatric units, as well as in the Emergency Care Unit (ECU), surgical suite, or adult, neonatal, and pediatric Intensive Care Units (ICUs) participated in the study. Eligible participants were nurses with at least six months of professional experience at the hospital. Professionals with ethical or clinical impediments that could compromise participation, such as cognitive difficulties interfering with comprehension of the instruments or explicit refusal to participate, were excluded. Nurses who met the inclusion criteria but were on sick leave, maternity leave, or vacation during data collection were considered sample losses. Convenience sampling was employed.

The sample size was calculated using Epi Info 6.04, considering a population of 183 nurses, a 5% margin of error, and a 95% confidence level, resulting in an estimated sample of 125 participants. The final sample, however, consisted of 124 nurses due to one professional’s sick leave. The difference of only one participant represents less than 1% of the estimated sample and does not compromise representativeness, statistical validity, or reliability of the results, keeping the technical parameters within acceptable limits. The STROBE (Strengthening the Reporting of Observational Studies in Epidemiology) checklist was used to ensure greater methodological rigor.

### Data collection

Data collection was conducted between September and December 2024, after the study objectives and procedures had been explained to the participants who were invited by the lead researcher. All participants completed a self-administered questionnaire, which took approximately 40 to 45 minutes to complete.

A questionnaire was used to characterize the sample, addressing sociodemographic variables (age, sex, race/ethnicity, marital status, family income, education, self-perception of physical health, self-perception of mental health, presence of clinical pathology, presence of psychiatric pathology) and occupational variables (weekly working hours, length of professional activity, work shift, employment relationship, primary sector of activity, and leadership role). The instrument was adapted from the model proposed by Morais and Silva^(19)^.

The dependent variable, corresponding to the level of occupational stress, was measured using the Brazilian version of the Work Stress Scale (WSS), validated by Paschoal and Tamayo^(20)^. This instrument assesses how frequently workers experience stressful situations in the workplace. The scale consists of items rated on a five-point Likert scale, ranging from 1 (never) to 5 (always).

The Work Coping Strategies Inventory (WCSI), validated for the Brazilian context by Savóia, Santana, and Mejias^(21)^, was used as an independent variable. It consists of eight domains that represent different coping strategies: Confrontation, Withdrawal, Self-Control, Social Support, Acceptance of Responsibility, Escape and Avoidance, Problem Solving and Positive Reappraisal. The items were assessed using a four-point Likert scale, ranging from 0 (never) to 3 (often).

Prior to data collection, a pilot study was conducted with 10 nurses from the same institution to assess the clarity of the instruments, average response time, and suitability of the questions for the hospital context. The pilot study participants were retained in the final sample, as no substantive changes to the instruments were necessary after this stage. The observations obtained led to minor language adjustments to the sociodemographic questionnaire, without altering the structure of the validated scales. The pilot study ensured that the data collection process proceeded as expected and that participants clearly understood the items.

### Data Analysis

Data were organized in Microsoft Excel^®^ spreadsheets and subsequently analyzed using the Statistical Package for the Social Sciences (SPSS), version 27.0. For the Work Stress Scale, the scores obtained for each item were summed and standardized as percentages from 0% to 100%, and then classified as low, moderate, or high stress levels. For the Work Coping Strategies Inventory, responses were grouped according to its eight theoretical domains, and mean scores were calculated for each domain to identify the predominant coping strategies. Sociodemographic and occupational variables were classified as either quantitative or categorical.

Descriptive statistical analyses included frequencies, means, and standard deviations, as well as association tests between variables, as appropriate. Quantitative variables were described using means and standard deviations or medians and interquartile ranges, while categorical variables were summarized by absolute and relative frequencies. Student’s t-test or Analysis of Variance (ANOVA), followed by Tukey’s post hoc test, was used to compare means. Associations between numerical variables were determined using Pearson’s linear correlation test, and those between categorical variables were determined using Pearson’s Chi-square test or Fisher’s exact test. Poisson regression was employed to control for confounding factors and identify independent associations between variables. Variables with p <0.20 in the bivariate analysis were included in the multivariate model, and those with p <0.05 remained in the final model. The level of significance was set at 5% (p<0.05).

## RESULTS

The study included 124 nurses, with a mean age of 36.6 years (± 7.9), indicating a relatively young workforce (Table 1). There was a predominance of women (74.2%), consistent with the historical feminization of nursing. Most participants (58.1%) reported a family income between five and ten times the minimum wage, suggesting a stable economic condition. The mean weekly workload was 36 hours (± 3.0), with a predominance of day-shift work (58.9%). Notably, 40.3% had been employed at the institution for 10 years or longer, reflecting long-term professional ties. A minority of nurses (10.5%) held managerial positions, indicating a concentration of direct care activities among participants.

**Table 1:** Characterization of the sample, Rio Grande, Rio Grande do Sul, Brazil, 2025 (N = 124)

Variables^*^	N = 124
Age (years) - mean ± SD	36.6 ± 7.9
Sex - n(%)	
Male	32 (25.8)
Female	92 (74.2)
Family income - n(%)	
3 to 5 times the MW	38 (30.6)
5 to 10 times the MW	72 (58.1)
10 to 20 times the MW	14 (11.3)
Weekly working hours - mean ± SD	36.0 ± 3.0
Time of experience - n (%)	
6 to 12 months	7 (5.6)
1 to 5 years	34 (27.4)
6 to 9 years	33 (26.6)
10 years or more	50 (40.3)
Work shift - n(%)	
Day shift	73 (58.9)
Night shift	51 (41.1)
Currently holds a management, leadership or coordination role - n(%)	
Yes	13 (10.5)
No	111 (89.5)

*MW - Minimum Wage; R$1,518.00 ≈ USD 267.10 (1 USD = R$5.68)*

**Figure 1:**
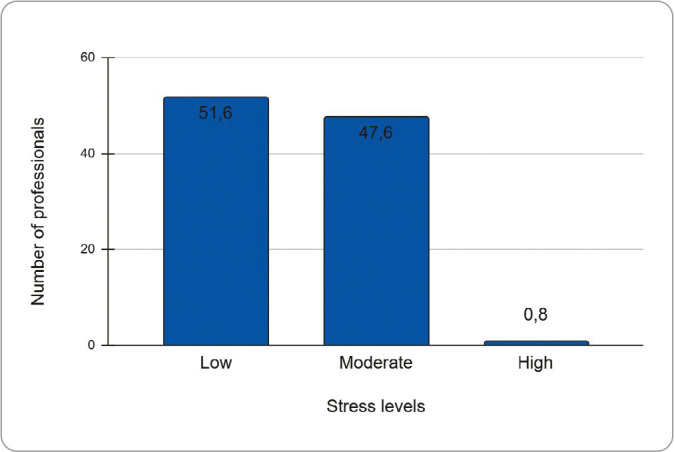
Stress levels according to the number of nurses, Rio Grande, Rio Grande do Sul, Brazil, 2025 (N = 124)

The analysis of stress levels indicated that more than half of the nurses (51.6%) had low levels of stress, followed by a significant proportion with moderate stress levels (47.6%). Only 0.8% of the participants reported high levels of stress, as shown in Figure 1.

The descriptive statistics for coping strategies scores in the total sample and by stress level are presented in Table 2. The domain with a significantly higher mean score was Escape and Avoidance among nurses with the highest stress levels (p<0.001).

**Table 2:** Associations between stress levels and coping domains, Rio Grande, Rio Grande do Sul, Brazil, 2025 (N=124)

^*^Domains	Total sample (n=124)	Low stress level (n = 64)	Moderate/high stress level (n = 60)	*p* value
	Mean ± SD	Mean ± SD	Mean ± SD	
**Confrontation**	0.92 ± 0.50	0.87 ± 0.49	0.98 ± 0.52	0.247
**Withdrawal**	1.01 ± 0.48	0.93 ± 0.49	1.09 ± 0.47	0.062
Self-Control	1.39 ± 0.52	1.33 ± 0.53	1.44 ± 0.51	0.244
Social Support	1.41 ± 0.61	1.33 ± 0.57	1.49 ± 0.64	0.123
Acceptance of Responsibility	1.31 ± 0.64	1.25 ± 0.63	1.37 ± 0.64	0.279
**Escape and Avoidance**	0.79 ± 0.56	0.63 ± 0.56	0.97 ± 0.52	<0.001
Problem-Solving	1.50 ± 0.62	1.49 ± 0.66	1.51 ± 0.58	0.811
Positive Reappraisal	1.40 ± 0.63	1.36 ± 0.65	1.45 ± 0.61	0.415

* *Bold - negative domains; SD - Standard Deviation.*

Table 3 presents the associations between stress levels and sociodemographic and occupational variables. A statistically significant association was found only between current self-perceived mental health and stress levels (p=0.013). Nurses with low stress levels reported better self-perceived mental health, whereas those with moderate or high stress levels were proportionally more likely to report poor/very poor self-perceived mental health.

**Table 3:** Associations between stress levels and sociodemographic and occupational variables, Rio Grande, Rio Grande do Sul, Brazil, 2025 (N = 124)

Variables	Low level of stress (n = 64)	Moderate/high level of stress (n = 60)	*p* value
Age (years) - mean ± SD	36.8 ± 7.9	36.4 ± 8.1	0.781
Sex - n(%)			0.215
Male	13 (20.3)	19 (31.7)	
Female	51 (79.7)	41 (68.3)	
Marital status - n(%)			1.000
With partner	25 (39.1)	23 (38.3)	
Without partner	39 (60.9)	37 (61.7)	
Family income - n(%)			0.720
3 to 5 times the MW	19 (29.7)	19 (31.7)	
5 to 10 times the MW	39 (60.9)	33 (55.0)	
10 to 20 times the MW	6 (9.4)	8 (13.3)	
Weekly working hours - mean ± SD	36.1 ± 2.7	35.7 ± 3.3	0.470
Time of experience - n(%)			0.653
6 to 12 months	5 (7.8)	2 (3.3)	
1 to 5 years	18 (28.1)	16 (26.7)	
6 to 9 years	15 (23.4)	18 (30.0)	
10 years or more	26 (40.6)	24 (40.0)	
Work shift - n(%)			0.078
Day shift	43 (67.2)	30 (50.0)	
Night shift	21 (32.8)	30 (50.0)	
Most prevalent workplace - n(%)			0.865
Medical clinic	14 (21.9)	13 (21.7)	
Neonatal/Pediatric ICU	8 (12.5)	6 (10.0)	
Surgical suite	5 (7.8)	6 (10.0)	
Emergency Care Unit (ECU)	6 (9.4)	4 (6.7)	
Maternity	4 (6.3)	5 (8.3)	
Traumatology	2 (3.1)	5 (8.3)	
Currently holds a management, leadership, or coordination role - n(%)	8 (12.5)	5 (8.3)	0.643
Current self-perceived physical health - n(%)			0.486
Excellent/Good	44 (68.8)	36 (60.0)	
Moderate	18 (28.1)	20 (33.3)	
Poor/Very poor	2 (3.1)	4 (6.7)	
Current self-perceived mental health - n(%)			0.013
Excellent/Good	44 (68.8)^*^	27 (45.0)	
Moderate	18 (28.1)	25 (41.7)	
Poor/Very poor	2 (3.1)	8 (13.3)^*^	
Have a psychiatric illness - n(%)			0.169
Yes	3 (4.7)	8 (13.3)	
No	61 (95.3)	52 (86.7)	

* *statistically significant association verified by the adjusted residuals test at the 5% significance level; SD - Standard Deviation; MW - Minimum Wage.*

Table 4 presents the results of the multivariate model, showing that the variables remaining significantly associated with higher stress levels were the Escape-Avoidance coping strategy (p=0.008) and poor/very poor self-perceived mental health (p=0.006). For each additional point in the Escape-Avoidance score, there was a 49% increase in the prevalence of moderate/high stress levels. Among nurses with poor or very poor current self-perceived mental health, the prevalence of moderate/high stress was 80% higher compared to those with excellent/good self-perception. The other variables included in the model were not statistically significant.

**Table 4:** Poisson regression analysis of factors independently associated with moderate or high stress levels.^*^

Variables	Prevalence Ratio	95% CI	*p* value
Coping strategies			
Escape and Avoidance	**1.49**	**1.11 - 2.00**	**0.008**
Current self-perceived mental health			
Excellent/Good	1.00	-	-
Moderate	1.43	0.98 - 2.09	0.067
Poor/Very poor	**1.80**	**1.18 - 2.75**	**0.006**
Have at least one psychiatric condition	1.16	0.75 - 1.79	0.509
Night shift	1.34	0.95 - 1.89	0.095
Coping strategies			
Withdrawal	0.97	0.62 - 1.52	0.899
Social Support	0.97	0.68 - 1.37	0.853

*IC95% - Intervalo de Confiança de 95%.*

## DISCUSSION

The results of this study contribute to a better understanding of occupational stress among nurses and the coping strategies used to address work demands in relation to sociodemographic and occupational variables. Although most nurses exhibited low stress levels, moderate and high levels were also identified, indicating that the phenomenon remains relevant in their professional routine. Evidence from the literature shows that factors such as work overload, lack of resources, limited institutional support, and high emotional demands are among the main determinants of stress in nursing. These factors have been associated with increased stress levels in hospital settings characterized by excessive workload and adverse working conditions^(12-22)^. Moreover, stress may vary according to length of professional experience and the accumulation of roles, which increase exposure to emotionally demanding situations and contribute to burnout^(13-23)^.

A potential explanation for the divergent stress level data observed in this study, compared with the literature^(12,13,23)^, relates to the sample profile. Most participants worked a regulated 36-hour work week, had a stable work relationship, and had been employed for 10 years or more, suggesting adaptation to the work routine and accumulated experience. Furthermore, only 10.5% held management positions, a role traditionally associated with greater emotional overload. The fact that the study was conducted in a public university hospital, characterized by stronger institutional support, a more stable environment, and encouragement of professional qualifications, may have acted as a protective factor against occupational stress. This context differs from studies^(4,6,7,10)^ conducted in highly complex or private healthcare settings.

Increased stress levels have also been associated with aging among professionals, attributed to the cumulative effect of burnout in nursing^(2-24)^. This finding differs from the results of the present study, whose sample had a mean age of 36 years. However, even in a healthcare institution with a positive organizational culture, such as that of this study, considerable moderate to high stress levels were observed among nurses. Research indicates that, regardless of age, exposure to high emotional demands and work overload can sustain or exacerbate occupational stress^(25,26)^, reinforcing the importance of recognizing and monitoring this phenomenon within the institution where this study was conducted.

Regarding coping strategies, the analysis revealed that nurses with moderate to high levels of stress more frequently resorted to the Escape and Avoidance strategy, indicating a pattern of avoidant coping with work demands. Studies show that, in the context of nurses’ behavioral responses to stress, this strategy is characterized by attempts to avoid or minimize contact with situations perceived as threatening or emotionally draining, such as interpersonal conflicts, critical decisions, and care overload. It represents a defense mechanism activated in work environments marked by scarce resources, high demands, institutional insecurity, and lack of psychosocial support, factors that hinder active coping with adversity. Although it may provide momentary relief, it does not address the underlying causes of stress and, in the long term, tends to exacerbate psychological distress^(6,7,10,13,14)^. Moreover, studies conducted during the COVID-19 pandemic confirm the predominance of this coping pattern among healthcare professionals exposed to high levels of uncertainty and emotional pressure^(27-29)^, reinforcing the relevance of institutional strategies that promote more adaptive coping mechanisms.

Despite this, other strategies, such as problem-solving, social support, and positive reappraisal, showed higher mean scores, although without statistically significant differences between the groups analyzed. These findings suggest that such strategies are part of nurses’ daily routines but may not be sufficient to neutralize the effects of more intense stress. Previous studies have identified social support as an important protective factor for mental health, helping to mitigate the negative impacts of occupational stress^(30,31)^. In the literature^(32)^, these strategies are defined as forms of active coping, as they involve actions aimed at addressing problems and regulating their emotional impact. The effectiveness of active coping strategies, such as self-control, problem-solving, and seeking support, depends on the strengthening of institutional aspects, including active listening, continuing education, and the creation of collective care spaces in the workplace.

A statistically significant association was found between self-perceived mental health and stress levels, indicating that nurses with lower stress levels tend to perceive their mental health more positively. Studies show that self-perceived mental health reflects an individual’s subjective evaluation of their psychological and emotional state, encompassing aspects such as balance, well-being, and ability to cope with daily demands. Therefore, environments with lower stress levels are associated with better perceptions of mental health^(11,33)^. Conversely, high stress levels contribute to poorer perceptions of mental health and are recognized as a risk factor for psychological distress^(7)^.

The analysis also identified a 49% increase in the prevalence of the Escape-Avoidance coping strategy among nurses with moderate or high stress levels. Studies^(34,35)^ indicate that when exposed to constant pressure, many professionals adopt avoidance strategies as a form of self-protection, such as withdrawing from difficult decisions, avoiding communication with colleagues, or neglecting patients’ emotional needs. Although this type of coping may provide temporary relief, it does not address the underlying stressors and, over time, tends to exacerbate psychological distress. Avoidance strategies have been associated with greater anxiety, emotional exhaustion, and a higher risk of burnout, reinforcing the importance of promoting more functional and adaptive coping strategies in daily nursing practice.

Finally, regarding self-perceived mental health, nurses who rated their mental health as poor or very poor were 80% more likely to report high stress levels compared to those with excellent or good self-perceptions. According to the literature, self-perceived mental health is a sensitive indicator that can reflect the impact of working conditions on well-being at an early stage, reinforcing the close relationship between occupational stress and professionals’ mental health. Consequently, nurses with poorer self-perceived mental health are more vulnerable to psychosocial problems, particularly when working under conditions of overload, long or exhausting work hours, lack of recognition, and insufficient institutional support. In everyday nursing practice, these professionals frequently face emotionally demanding situations, such as dealing with death, patient suffering, and staff shortages, without adequate spaces for support or active listening, which contributes to the worsening of chronic stress^(5,36)^.

### Study limitations

This cross-sectional study was conducted in a single hospital, which limits the ability to infer causal relationships between the variables analyzed. Additionally, the findings are based on nurses’ self-reported perceptions, which are inherently subjective. Nonetheless, standardized and validated instruments were used to minimize potential bias. To enhance the generalizability of results, future longitudinal studies using random sampling are recommended.

### Contributions to the nursing, health, and public policy

This study makes a meaningful contribution to nursing, occupational health, and public policy. In the field of nursing, it underscores the importance of institutional initiatives that promote psychosocial care, active listening, and the use of adaptive coping strategies to manage stress. The findings also support the advancement of public policies on occupational health within the Unified Health System (SUS), particularly regarding the implementation of emotional support programs, mental health training, and the promotion of healthier and more sustainable work environments. Finally, the evidence obtained contributes to improving management practices and developing preventive interventions in hospital settings.

## CONCLUSIONS

This study examined the frequency of stress among nurses, ranging from low to moderate and high levels. The findings revealed that nurses with higher stress levels more frequently used Escape and Avoidance coping strategies, indicating the adoption of avoidant mechanisms when faced with work demands. Conversely, nurses with low stress levels reported better self-perceived mental health.

These results contribute to a deeper understanding of coping dynamics within the context of nurses’ work-related stress. Above all, they highlight the importance of comprehensive institutional policies aimed at professional development, strengthening interpersonal relationships, promoting active listening, and restructuring organizational working conditions. Therefore, promoting nurses’ mental health requires structural and collective changes that enable more effective coping with occupational stress and its impacts on care delivery.

The study concludes that recognizing stressors, identifying coping patterns, and implementing public policies that prioritize nurses’ mental health are essential to fostering healthier, more collaborative, and sustainable work environments. The findings support the development of institutional programs and the strengthening of comprehensive occupational health care guidelines within the Unified Health System (SUS), aligned with contemporary demands for high-quality care and the valuing of nursing professionals.

## Data Availability

The research data are available only upon request.
