# Supplementary material for: Stress in hospital nurses: association between coping strategies and sociodemographic and occupational variables
Source: Rev Bras Enferm. 2026 Jul 6;79:e20250258. doi: 10.1590/0034-7167-2025-0258 (PMC13336231; doi:10.1590/0034-7167-2025-0258)
Supplement: Translation

# Translation: Estresse em enfermeiros hospitalares: associação entre estratégias de *coping* e variáveis sociodemográficas e ocupacionais

**DOI:** 10.1590/0034-7167-2025-0258pt

**Authors:** Manuela Ribeiro dos Santos, Daiani Modernel Xavier

**Affiliations:** I Universidade Federal do Rio Grande. Rio Grande, Rio Grande do Sul, Brasil

**Keywords:** Estresse Psicológico, Adaptação Psicológica, Relações Interpessoais, Saúde Ocupacional, Enfermagem.

## Abstract

**Objetivos:**

avaliar a associação entre o nível de estresse de enfermeiros hospitalares, as estratégias de *coping* utilizadas, e as variáveis sociodemográficas e ocupacionais.

**Métodos:**

estudo descritivo, transversal e de abordagem quantitativa, realizado com 124 enfermeiros de hospital universitário no sul do Brasil. Utilizaram-se instrumentos autoaplicáveis, como questionário sociodemográfico, Escala de Estresse no Trabalho e Inventário de Respostas de *Coping* no Trabalho. A análise de dados foi realizada por meio de estatística descritiva e inferencial.

**Resultados:**

verificaram-se níveis de estresse em nível baixo de estresse com proximidade aos níveis médio e alto. Enfermeiros com estresse elevado utilizaram a estratégia de *coping* do tipo fuga e esquiva, enquanto aqueles com baixo estresse relataram melhor autopercepção da saúde mental.

**Conclusões:**

os achados contribuem para subsidiar programas institucionais e reforçar diretrizes de atenção integral à saúde do trabalhador no Sistema Único de Saúde.

## INTRODUÇÃO

O trabalho do enfermeiro no âmbito hospitalar se caracteriza por intensa complexidade, envolvendo múltiplas demandas técnicas, emocionais e organizacionais, que exigem dos profissionais um contínuo processo de adaptação e resposta a situações de alta pressão^(1,2)^. Essa realidade torna a profissão particularmente suscetível ao estresse ocupacional, decorrente da interação entre as exigências do ambiente de trabalho e a percepção dos recursos individuais disponíveis para enfrentá-las^(3-5)^. Nesse contexto, as estratégias de *coping*, entendidas como esforços cognitivos e comportamentais para adaptação às adversidades, assumem papel central, podendo atenuar ou intensificar os efeitos negativos do estresse, a depender de sua natureza adaptativa ou disfuncional^(6,7)^.

A natureza relacional e assistencial da profissão, aliada à exposição frequente a cenários de sofrimento humano, morte e limitações institucionais, contribui para o desenvolvimento do estresse ocupacional^(3)^. Esse fenômeno, quando não manejado de forma adequada, compromete não apenas a saúde física e mental dos trabalhadores, mas também a qualidade do cuidado prestado à população.

O estresse ocupacional se configura como fenômeno multifatorial resultante da interação entre as exigências do ambiente de trabalho e a percepção dos profissionais sobre os recursos individuais disponíveis para enfrentá-las^(4,5)^. No campo da enfermagem, destacam-se as estratégias de *coping* como mecanismos destinados à gestão do estresse. Estratégias consideradas adaptativas, como resolução de problemas, autocontrole emocional e busca de suporte social, demonstram eficácia na mitigação dos efeitos negativos do estresse e no fortalecimento da resiliência. Em contrapartida, o uso recorrente de estratégias disfuncionais, como fuga, esquiva e afastamento emocional, tende a intensificar o sofrimento psíquico e aumentar a vulnerabilidade ao adoecimento^(6,7)^.

No enfrentamento cotidiano das pressões e demandas do trabalho, o enfermeiro pode manifestar comportamentos como retraimento social, irritabilidade, desmotivação, procrastinação, cinismo e conflitos interpessoais entre profissionais de enfermagem. Tais manifestações refletem estratégias de enfrentamento ineficazes diante do estresse ocupacional, contribuindo para perpetuar esse cenário. Quando não abordados adequadamente, esses comportamentos comprometem o clima organizacional, fragilizam a coesão das equipes e impactam negativamente a segurança do paciente, configurando um importante problema de saúde coletiva no contexto hospitalar^(8)^.

No contexto dos serviços de saúde, o enfermeiro frequentemente enfrenta ambientes de trabalho marcados por hostilidade, sobrecarga e elevados níveis de estresse. Essa realidade está associada ao aumento de condições como *burnout*, sintomas depressivos, absenteísmo e intenção de desligamento da profissão^(9)^. Diante desse cenário, torna-se essencial que as instituições de saúde implementem ações voltadas à promoção de ambientes laborais saudáveis, ao estímulo de estratégias de enfrentamento positivas e à identificação precoce de sinais de sofrimento psíquico como parte integrante das políticas de cuidado com os trabalhadores^(10)^.

Além disso, intervenções organizacionais, que combinam estratégias de suporte emocional, capacitação em estratégias de *coping* adaptativo e fortalecimento de redes de apoio social, apresentam resultados promissores na redução do estresse e na promoção da saúde mental dos trabalhadores. Programas estruturados de suporte emocional no ambiente de trabalho contribuem para redução da prevalência de estresse e aumento do bem-estar psicológico^(11,12)^. Essas intervenções repercutem diretamente na qualidade do cuidado oferecido e na sustentabilidade dos serviços de saúde.

Torna-se fundamental compreender que o modo como os enfermeiros enfrentam o estresse ocupacional não é uniforme, pois reflete uma complexa interação entre fatores individuais e contextuais. Aspectos sociodemográficos, como idade, sexo, estado civil e escolaridade, e características ocupacionais, como jornada semanal, tempo de atuação profissional, setor de trabalho e tipo de vínculo empregatício, exercem influência direta sobre a percepção do estresse e a escolha das estratégias de *coping* ^(13-15)^. Esses fatores funcionam como determinantes sociais da saúde do trabalhador, e devem ser considerados de forma crítica, uma vez que contribuem para configurar diferentes perfis de vulnerabilidade psicossocial nas instituições hospitalares^(16)^. Assim, reconhecer essas variáveis se mostra essencial para formulação de intervenções equitativas e eficazes no campo da saúde mental e na promoção de ambientes de trabalho saudáveis.

Diante da complexidade e da magnitude do tema, torna-se fundamental aprofundar a compreensão das interações entre estresse ocupacional, estratégias de *coping* e percepção da saúde mental no exercício da enfermagem. Reconhecer essas relações é essencial para subsidiar práticas gerenciais, políticas institucionais e ações voltadas à prevenção do adoecimento psíquico. Este estudo se mostra relevante ao evidenciar padrões de enfrentamento e seus efeitos sobre o bem-estar dos trabalhadores, contribuindo para o fortalecimento de intervenções no campo da saúde do trabalhador.

Nesse sentido, este estudo propõe-se a responder à seguinte questão norteadora: como o nível de estresse entre enfermeiros hospitalares se associa às estratégias de *coping* e às variáveis sociodemográficas e ocupacionais?

## OBJETIVOS

Avaliar a associação entre o nível de estresse de enfermeiros hospitalares, as estratégias de *coping* utilizadas, e as variáveis sociodemográficas e ocupacionais.

## MÉTODOS

### Aspectos éticos

A pesquisa foi aprovada pelo Comitê de Ética em Pesquisa, conforme a Resolução n^o^ 466/2012 do Conselho Nacional de Saúde^(17)^. A coleta de dados teve início após aprovação ética e anuência institucional. Em respeito ao princípio da autonomia, todos os participantes foram esclarecidos sobre os procedimentos, e assinaram o Termo de Consentimento Livre e Esclarecido em duas vias, sendo uma entregue ao participante.

### Desenho do estudo e plano amostral

Trata-se de pesquisa descritivo-exploratória, de corte transversal, com abordagem quantitativa. Este delineamento busca descrever e aprofundar o conhecimento sobre fenômenos ainda pouco explorados, observando a população em um único ponto no tempo, e utilizando a análise estatística para identificar padrões e relações^(18)^. O estudo foi desenvolvido em hospital universitário público, vinculado ao Sistema Único de Saúde (SUS), localizado no extremo sul do Brasil. A instituição é referência regional para atendimentos de média e alta complexidade, possuindo uma ampla estrutura assistencial. Participaram da pesquisa enfermeiros atuantes nas unidades de clínica médica, clínica cirúrgica, traumatologia, maternidade, clínica pediátrica, Serviço de Pronto Atendimento, bloco cirúrgico, e Unidades de Terapia Intensiva Adulto, Neonatal e Pediátrica. Foram incluídos enfermeiros com tempo mínimo de atuação de seis meses no hospital. Foram excluídos da pesquisa os profissionais que apresentavam impedimentos éticos ou clínicos capazes de inviabilizar a participação, como dificuldades cognitivas que prejudicassem a compreensão dos instrumentos ou recusa explícita em participar. Já os profissionais que, embora elegíveis, estavam afastados por licença-saúde, licença-maternidade ou férias no período da coleta de dados foram considerados como perdas amostrais. Foi utilizada uma amostragem por conveniência.

O cálculo amostral foi realizado por meio do programa *Epi Info* 6.04, considerando uma população de 183 enfermeiros, margem de erro de 5% e nível de confiança de 95%, resultando em uma amostra estimada de 125 participantes. Contudo, a amostra final foi composta por 124 enfermeiros, em razão de licença à saúde de um profissional. Ressalta-se que essa diferença de apenas um participante representa menos de 1% da amostra estimada, e não exige a representatividade, validade estatística ou confiabilidade dos resultados obtidos, mantendo-se as especificações técnicas dentro dos limites aceitáveis. O instrumento norteador STROBE, ou *STrengthening the Reporting of OBservational studies in Epidemiology*, foi utilizado para assegurar maior rigor metodológico à pesquisa.

### Coleta de dados

A coleta de dados realizou-se entre os meses de setembro e dezembro de 2024, após esclarecimento dos objetivos e procedimentos da pesquisa aos participantes, que foram convidados pelo pesquisador responsável. Todos os respondentes preencheram um questionário autoaplicável, com tempo médio estimado de 40 a 45 minutos.

Para caracterização da amostra, utilizou-se instrumento que contemplou variáveis sociodemográficas (idade, sexo, cor da pele, estado civil, renda familiar, escolaridade, autopercepção da saúde física, autopercepção da saúde mental, presença de patologia clínica e presença de patologia psiquiátrica) e laborais (horas de trabalho semanal, tempo de atuação profissional, turno de trabalho, vínculo empregatício, setor de atuação mais prevalente e função de liderança), adaptado do modelo proposto por Morais e Silva^(19)^.

A variável dependente, correspondente ao nível de estresse ocupacional, foi mensurada por meio da Escala de Estresse no Trabalho (EET), validada por Paschoal e Tamayo^(20)^. Trata-se de instrumento validado que avalia a frequência com que os trabalhadores vivenciam situações geradoras de estresse no ambiente laboral. A escala é composta por itens avaliados em escala do tipo Likert de cinco pontos, com variação de 1 (nunca) a 5 (sempre).

Como variável independente, utilizou-se o Inventário de Respostas de *Coping* no Trabalho (IRC-T), adaptado para o contexto brasileiro por Savóia, Santana e Mejias^(21)^, composto por oito domínios que representam diferentes estratégias de enfrentamento, como confronto, afastamento, autocontrole, suporte social, aceitação de responsabilidade, fuga e esquiva, resolução de problemas, e reavaliação positiva. Os itens foram avaliados por meio de escala Likert de quatro pontos, variando de 0 (nunca) a 3 (frequentemente).

Antes da coleta principal, foi conduzido estudo piloto com dez enfermeiros da própria instituição, com o objetivo de avaliar a clareza dos instrumentos, tempo médio de resposta e adequação das questões ao contexto hospitalar. Os participantes do estudo piloto foram mantidos na amostra final, uma vez que não houve necessidade de alterações substantivas nos instrumentos após essa etapa. As observações obtidas serviram para ajustes mínimos de linguagem no questionário sociodemográfico, sem modificações na estrutura das escalas validadas. O piloto contribuiu para garantir as previsões do processo de coleta e compreensão dos itens por parte dos entrevistados.

### Análise de dados

Os dados foram organizados em planilhas do *Microsoft Excel^®^* e, posteriormente, analisados com o auxílio do *software* SPSS, ou *Statistical Package for the Social Sciences*, versão 27.0. Para a EET, procedeu-se à soma dos escores obtidos em cada item, com posterior padronização em percentuais de 0% a 100%, classificados em níveis de estresse baixo, moderado ou alto. Quanto ao IRC-T, as respostas foram agrupadas conforme os oito domínios teóricos propostos, calculando-se a média das pontuações de cada domínio para identificação das estratégias de enfrentamento predominantes. Além disso, as variáveis sociodemográficas e laborais foram agrupadas em variáveis quantitativas e variáveis categóricas.

As análises estatísticas descritivas incluíram frequências, médias e desvios padrão, além de testes de associação entre variáveis, conforme a necessidade da investigação. As variáveis quantitativas foram descritas por média e desvio padrão ou mediana e amplitude interquartílica. As variáveis categóricas foram descritas por frequências absolutas e relativas. Para comparar médias, os testes t de Student ou Análise de Variância, complementados pelo teste de Tukey, foram aplicados. A associação entre variáveis numéricas foi realizada pelo teste de correlação linear de Pearson, e entre variáveis categóricas, pelos testes qui-quadrado de Pearson ou exato de Fisher. Para controle de fatores de confusão e identificação de associações independentes entre as variáveis, utilizou-se a regressão de Poisson. Foram incluídas no modelo multivariado as variáveis que apresentaram valor de p < 0,20 na análise bivariada, permanecendo no modelo final aquelas com p < 0,05. O nível de significância adotado foi de 5% (p<0,05).

## RESULTADOS

Participaram do estudo 124 enfermeiros, com idade média de 36,6 anos (± 7,9), caracterizando uma força de trabalho relativamente jovem (Tabela 1). Observou-se predominância do sexo feminino (74,2%), perfil condizente com a feminilização histórica da enfermagem. A maior parte dos profissionais (58,1%) possuía renda familiar entre cinco e dez salários mínimos, o que sugere inserção em nível econômico estável. A jornada semanal média foi de 36 horas (± 3,0), com prevalência de atuação no turno diurno (58,9%). Destaca-se que 40,3% atuavam na instituição há dez anos ou mais, indicando vínculos duradouros com o serviço. Uma minoria exerce cargos de gestão (10,5%), evidenciando concentração da atuação assistencial direta entre os participantes.

**Tabela 1:** Caracterização da amostra dos enfermeiros, Rio Grande, Rio Grande do Sul, Brasil, 2025 (N = 124)

Variáveis^*^	N = 124
Idade (anos) - média ± DP	36,6 ± 7,9
Sexo - n(%)	
Masculino	32 (25,8)
Feminino	92 (74,2)
Renda familiar - n(%)	
>três a cinco s.m.	38 (30,6)
>cinco a dez s.m.	72 (58,1)
>dez a 20 s.m.	14 (11,3)
Horas de trabalho semanal - média ± DP	36,0 ± 3,0
Tempo de atuação - n(%)	
Seis a 12 meses	7 (5,6)
Um a cinco anos	34 (27,4)
Seis a nove anos	33 (26,6)
Dez anos ou mais	50 (40,3)
Turno de trabalho - n(%)	
Diurno	73 (58,9)
Noturno	51 (41,1)
Exerce atualmente funções de direção, chefia ou coordenação - n(%)	
Sim	13 (10,5)
Não	111 (89,5)

*s.m. - salário mínimo R$1.518,00 ≈ USD 267,10 (1 USD = R$5,68); DP - desvio padrão.*

Na análise dos níveis de estresse, constatou-se que mais da metade dos enfermeiros (51,6%) apresentou estresse em nível baixo, seguida por uma proporção expressiva com estresse moderado (47,6%). Apenas 0,8% dos participantes relataram nível alto de estresse, conforme mostrado na Figura 1.

**Figura 1:** Nível de estresse conforme o número de enfermeiros, Rio Grande, Rio Grande do Sul, Brasil, 2025 (N = 124)

As estatísticas descritivas na amostra total e por nível de estresse dos escores das estratégias de *coping* estão apresentadas na Tabela 2. O domínio que apresentou média significativamente maior foi “fuga e esquiva” nos enfermeiros com mais alto nível de estresse (p<0,001).

**Tabela 2:** Associações dos níveis de estresse com os domínios de *coping*, Rio Grande, Rio Grande do Sul, Brasil, 2025 (N = 124)

^*^Domínios	Amostra total (n = 124)	Nível baixo de estresse (n = 64)	Nível médio/alto de estresse (n = 60)	Valor de *p*
	Média ± DP	Média ± DP	Média ± DP	
**Confronto**	0,92 ± 0,50	0,87 ± 0,49	0,98 ± 0,52	0,247
**Afastamento**	1,01 ± 0,48	0,93 ± 0,49	1,09 ± 0,47	0,062
Autocontrole	1,39 ± 0,52	1,33 ± 0,53	1,44 ± 0,51	0,244
Suporte social	1,41 ± 0,61	1,33 ± 0,57	1,49 ± 0,64	0,123
Aceitação de responsabilidade	1,31 ± 0,64	1,25 ± 0,63	1,37 ± 0,64	0,279
**Fuga e esquiva**	0,79 ± 0,56	0,63 ± 0,56	0,97 ± 0,52	<0,001
Resolução de problemas	1,50 ± 0,62	1,49 ± 0,66	1,51 ± 0,58	0,811
Reavaliação positiva	1,40 ± 0,63	1,36 ± 0,65	1,45 ± 0,61	0,415

* *Negrito - domínios negativos; DP - desvio padrão.*

A Tabela 3 demonstra os resultados da associação dos níveis de estresse com as variáveis sociodemográficas e laborais. Houve apenas a associação estatisticamente significativa da autopercepção de saúde mental atual com o nível de estresse (p = 0,013). Enfermeiros com baixo nível de estresse relatam melhor autopercepção de saúde mental do que aqueles com médio ou alto nível de estresse que apresentam, proporcionalmente, maior autopercepção de saúde ruim/muito ruim.

**Tabela 3:** Associações dos níveis de estresse com as variáveis sociodemográficas e laborais, Rio Grande, Rio Grande do Sul, Brasil, 2025 (N = 124)

Variáveis	Nível baixo de estresse (n = 64)	Nível médio/alto de estresse (n = 60)	Valor de *p*
Idade (anos) - média ± DP	36,8 ± 7,9	36,4 ± 8,1	0,781
Sexo - n(%)			0,215
Masculino	13 (20,3)	19 (31,7)	
Feminino	51 (79,7)	41 (68,3)	
Estado civil - n(%)			1,000
Com companheiro	25 (39,1)	23 (38,3)	
Sem companheiro	39 (60,9)	37 (61,7)	
Renda familiar - n(%)			0,720
>três a cinco s.m.	19 (29,7)	19 (31,7)	
>cinco a dez s.m.	39 (60,9)	33 (55,0)	
>dez a 20 s.m.	6 (9,4)	8 (13,3)	
Horas de trabalho semanal - média ± DP	36,1 ± 2,7	35,7 ± 3,3	0,470
Tempo de atuação - n(%)			0,653
Seis a 12 meses	5 (7,8)	2 (3,3)	
Um a cinco anos	18 (28,1)	16 (26,7)	
Seis a nove anos	15 (23,4)	18 (30,0)	
Dez anos ou mais	26 (40,6)	24 (40,0)	
Turno de trabalho - n(%)			0,078
Diurno	43 (67,2)	30 (50,0)	
Noturno	21 (32,8)	30 (50,0)	
Local de trabalho mais prevalentes - n(%)			0,865
Clínica médica	14 (21,9)	13 (21,7)	
Unidade de Terapia Intensiva Neonatal/Pediátrica	8 (12,5)	6 (10,0)	
Clínica cirúrgica	5 (7,8)	6 (10,0)	
Serviço de Pronto Atendimento	6 (9,4)	4 (6,7)	
Maternidade	4 (6,3)	5 (8,3)	
Traumatologia	2 (3,1)	5 (8,3)	
Exerce atualmente funções de direção, chefia ou coordenação - n(%)	8 (12,5)	5 (8,3)	0,643
Autopercepção de saúde física atual - n(%)			0,486
Excelente/boa	44 (68,8)	36 (60,0)	
Regular	18 (28,1)	20 (33,3)	
Ruim/muito ruim	2 (3,1)	4 (6,7)	
Autopercepção de saúde mental atual - n(%)			0,013
Excelente/boa	44 (68,8)^*^	27 (45,0)	
Regular	18 (28,1)	25 (41,7)	
Ruim/muito ruim	2 (3,1)	8 (13,3)^*^	
Tem alguma patologia psiquiátrica - n(%)			0,169
Sim	3 (4,7)	8 (13,3)	
Não	61 (95,3)	52 (86,7)	

* *associação estatisticamente significativa pelo teste dos resíduos ajustados a 5% de significância; DP - desvio padrão; s.m. - salário mínimo.*

A Tabela 4, após o ajuste pelo modelo multivariado, sintetiza que as variáveis que permaneceram significativamente associados a maiores níveis de estresse foram a estratégia de *coping* de “fuga e esquiva” (p = 0,008) e a autopercepção de saúde mental ruim/muito ruim (p = 0,006). Para um ponto a mais no escore de “fuga e esquiva”, há um aumento de 49% na prevalência de nível de estresse médio/alto. Para os enfermeiros com autopercepção de saúde mental atual ruim ou muito ruim, o aumento na prevalência de estresse médio/alto é de 80%, quando comparados com os que possuem autopercepção excelente/boa. As demais variáveis presentes no modelo não apresentaram significância estatística.

**Tabela 4:** Regressão de Poisson para avaliar fatores independentemente^*^ associados a níveis médios ou altos de estresse

Variáveis	Razão de Prevalência	IC95%	Valor de *p*
Estratégias de *coping*			
Fuga e esquiva	**1,49**	**1,11 - 2,00**	**0,008**
Autopercepção de saúde mental atual			
Excelente/boa	1,00	-	-
Regular	1,43	0,98 - 2,09	0,067
Ruim/muito ruim	**1,80**	**1,18 - 2,75**	**0,006**
Alguma patologia psiquiátrica	1,16	0,75 - 1,79	0,509
Turno noturno	1,34	0,95 - 1,89	0,095
Estratégias de *coping*			
Afastamento	0,97	0,62 - 1,52	0,899
Suporte social	0,97	0,68 - 1,37	0,853

*IC95% - Intervalo de Confiança de 95%.*

## DISCUSSÃO

Os resultados deste estudo contribuem para a compreensão do estresse ocupacional entre enfermeiros e das estratégias de *coping* utilizadas para enfrentar as demandas do trabalho associadas às variáveis sociodemográficas e ocupacionais. Embora a maioria apresentasse níveis baixos de estresse, foram também identificados níveis médios e altos, o que indica que o fenômeno permanece relevante na rotina profissional. Evidências da literatura mostram que fatores como sobrecarga de trabalho, deficiência de recursos, ausência de suporte institucional e altas demandas emocionais figuram entre os principais determinantes do estresse na enfermagem. Esses fatores têm sido associados a níveis mais elevados de estresse em contextos hospitalares marcados por sobrecarga e condições adversas^(12-22)^. Além disso, o estresse pode variar conforme o tempo de experiência e o acúmulo de funções, o que aumenta a exposição a situações emocionalmente desgastantes, favorecendo o esgotamento profissional^(13-23)^.

Uma possível justificativa para os dados divergentes nos níveis de estresse observados no presente estudo, em relação à literatura^(12,13,23)^, está relacionada ao perfil da amostra. A maioria dos participantes possuía jornada semanal regulamentada de 36 horas, vínculo institucional estável e tempo de atuação igual ou superior a dez anos, indicando adaptação à rotina laboral e acúmulo de experiência. Além disso, apenas 10,5% ocupavam cargos de chefia, função tradicionalmente associada à maior sobrecarga emocional. O fato de a pesquisa ter sido realizada em hospital universitário público, caracterizado por maior suporte institucional, ambiente mais estável e incentivo à qualificação profissional, pode ter atuado como fator protetivo diante do estresse ocupacional. Esse cenário difere de estudos^(4,6,7,10)^ realizados em contextos de alta complexidade ou rede privada.

O aumento dos níveis de estresse também foi associado ao envelhecimento dos profissionais, atribuído ao efeito cumulativo da exaustão entre enfermeiros^(2-24)^. Esse achado difere dos resultados deste estudo, cuja amostra apresentou média de idade de 36 anos. No entanto, mesmo em uma instituição de saúde com cultura organizacional positiva, como a deste estudo, observou-se considerável nível médio a alto de estresse entre esses profissionais. Pesquisas indicam que, independentemente da idade, a exposição a altas demandas emocionais e à sobrecarga de trabalho pode manter ou agravar o estresse ocupacional^(25,26)^, reforçando a importância de seu reconhecimento e monitoramento no contexto da pesquisa.

Em relação às estratégias de *coping*, a análise constatou que enfermeiros com níveis médios a altos de estresse recorreram com mais frequência à estratégia de “fuga e esquiva”, o que revelou um padrão de enfrentamento evitativo diante das exigências do trabalho. Estudos mostram que, no contexto do comportamento do enfermeiro como resposta ao estresse, essa estratégia se caracteriza pela tentativa de evitar ou minimizar o contato com situações percebidas como ameaçadoras ou emocionalmente desgastantes, como conflitos interpessoais, decisões críticas e sobrecarga assistencial. Trata-se de mecanismo de defesa acionado diante de contextos laborais marcados por escassez de recursos, exigências elevadas, insegurança institucional e ausência de suporte psicossocial, elementos que dificultam o enfrentamento ativo das adversidades. Embora possa proporcionar alívio momentâneo, não atua sobre as causas do estresse, tendendo, a longo prazo, a agravar o sofrimento psíquico^(6,7,10,13,14)^. Além disso, pesquisas realizadas durante a pandemia de COVID-19 confirmam a prevalência desse padrão de *coping* entre profissionais de saúde expostos a altos níveis de incerteza e pressão emocional^(27-29)^, reforçando a relevância de estratégias institucionais que favoreçam modos de enfrentamento mais adaptativos.

Apesar disso, outras estratégias, como resolução de problemas, suporte social e reavaliação positiva, apresentaram médias elevadas, ainda que sem diferença estatística significativa entre os grupos analisados. Esses achados indicam que tais estratégias fazem parte do cotidiano dos enfermeiros, mas possivelmente não neutralizam os efeitos do estresse mais intenso. Estudos anteriores apontaram o suporte social como um fator protetor importante para a saúde mental, contribuindo para reduzir os impactos negativos do estresse ocupacional^(30,31)^. Na literatura^(32)^, essas estratégias são definidas como formas de *coping* ativo, por envolver ações voltadas ao enfrentamento do problema e à regulação do impacto emocional. A efetividade de estratégias ativas de enfrentamento, como autocontrole, resolução de problemas e busca de apoio, depende do fortalecimento de aspectos institucionais, como a escuta qualificada, a educação permanente e a criação de espaços coletivos de cuidado no ambiente de trabalho.

Verificou-se uma associação estatisticamente significativa entre a autopercepção da saúde mental e os níveis de estresse, indicando que enfermeiros com menor nível de estresse tendem a perceber melhor sua saúde mental. Estudos referem que a autopercepção da saúde mental corresponde à avaliação subjetiva que o indivíduo faz sobre seu próprio estado psicológico e emocional, considerando aspectos como equilíbrio, bem-estar e capacidade de lidar com as demandas do cotidiano. Assim, ambientes com menor carga de estresse estão associados à melhor percepção da saúde mental^(11,33)^. Em contrapartida, níveis elevados de estresse contribuem para a pior percepção da saúde psíquica, sendo reconhecidos como fatores de risco para o sofrimento mental^(7)^.

A análise também identificou um aumento de 49% na prevalência do uso da estratégia de *coping* do tipo “fuga e esquiva” entre enfermeiros com níveis médios ou altos de estresse. Estudos^(34,35)^ mostram que, diante de pressões constantes, muitos profissionais recorrem a estratégias evitativas como forma de autoproteção, como afastar-se de decisões difíceis, evitar diálogos com colegas ou negligenciar demandas emocionais dos pacientes. Embora esse tipo de enfrentamento possa oferecer alívio imediato, ele não soluciona os fatores estressores e, a longo prazo, tende a intensificar o sofrimento mental. Estratégias evitativas estão associadas ao aumento da ansiedade, exaustão emocional e maior risco de *burnout*, o que reforça a necessidade de promover estratégias de enfrentamento mais funcionais e adaptativas no cotidiano da enfermagem.

Por fim, no que diz respeito à autopercepção da saúde mental, os enfermeiros que classificaram sua saúde mental como ruim ou muito ruim apresentaram 80% maior probabilidade de relatar níveis elevados de estresse, em comparação àqueles com percepção excelente ou boa. De acordo com a literatura, esse é um indicador sensível, capaz de refletir de forma precoce o impacto das condições laborais sobre o bem-estar, o que reforça a estreita relação entre estresse ocupacional e saúde mental dos profissionais. Nesse sentido, enfermeiros com pior saúde mental percebida estão mais expostos a agravos psicossociais, especialmente quando trabalham em contextos de sobrecarga, jornadas exaustivas, falta de reconhecimento e ausência de suporte institucional. No cotidiano da enfermagem, é comum que esses profissionais enfrentem situações emocionalmente desgastantes, como lidar com a morte, o sofrimento dos pacientes e a escassez de recursos humanos, sem espaços adequados para acolhimento ou escuta ativa, o que contribui para o agravamento do estresse crônico^(5,36)^.

### Limitações do estudo

Primeiro, foi estudo transversal em apenas um hospital, o que limita a capacidade de interpretar as relações causais entre as diferentes variáveis neste estudo. Além disso, os resultados apresentados provêm de estudo que se baseia na avaliação subjetiva dos sentimentos dos enfermeiros. No entanto, o estudo utilizou ferramentas de pesquisa padronizada. Para generalizar os resultados, futuros estudos longitudinais devem ser conduzidos usando amostragem aleatória.

### Contribuições para as áreas da enfermagem, saúde ou políticas públicas

O presente estudo contribui de forma relevante para a enfermagem, saúde ocupacional e políticas públicas. No campo da enfermagem, destaca-se a necessidade de ações institucionais que incentivem o cuidado psicossocial, escuta qualificada e uso de estratégias adaptativas para lidar com o estresse. Os resultados também oferecem subsídios para o aprimoramento das políticas públicas de saúde do trabalhador no âmbito do SUS, especialmente no que se refere à implementação de programas de apoio emocional, capacitação em saúde mental e promoção de ambientes de trabalho mais saudáveis e sustentáveis. Por fim, as evidências obtidas auxiliam na qualificação da gestão e no desenvolvimento de intervenções preventivas no contexto hospitalar.

## CONCLUSÕES

O presente estudo verificou a frequência dos níveis de estresse, em nível baixo de estresse, com proximidade aos níveis médio e alto, entre enfermeiros. Constatou-se, ainda, que enfermeiros com níveis mais elevados de estresse apresentaram maior utilização da estratégia de *coping* de “fuga e esquiva”, o que remete à adoção de mecanismos de enfrentamento evitativos perante as exigências laborais. Enfermeiros com níveis de estresse baixo tiveram melhor autopercepção da saúde mental.

Esses resultados contribuem para compreensão das dinâmicas de enfrentamento no contexto dos níveis de estresse no trabalho do enfermeiro. Evidenciam, sobretudo, a importância de políticas institucionais mais amplas de valorização profissional, fortalecimento das relações interpessoais, escuta qualificada e reestruturação das condições organizacionais de trabalho. Assim, a promoção da saúde mental dos enfermeiros exige mudanças estruturais e coletivas que permitam enfrentamento efetivo do estresse ocupacional e de seus impactos no cuidado.

Conclui-se que o reconhecimento dos fatores estressores, a identificação dos padrões de *coping* e a implementação de políticas públicas que priorizem a saúde mental dos enfermeiros são fundamentais para construção de ambientes de trabalho mais saudáveis, colaborativos e sustentáveis. Os achados deste estudo oferecem subsídios para desenvolvimento de programas institucionais e fortalecimento de diretrizes de atenção integral à saúde do trabalhador no SUS, alinhados às exigências contemporâneas de qualidade assistencial e valorização dos profissionais de enfermagem.

## Data Availability

Os dados de pesquisa só estão disponíveis mediante solicitação.
